# IL-6-174 G/C and -572 C/G Polymorphisms and Risk of Alzheimer’s Disease

**DOI:** 10.1371/journal.pone.0037858

**Published:** 2012-06-06

**Authors:** Hui-Ping Qi, Zheng-Yi Qu, Shu-Rong Duan, Shu-Qin Wei, Shi-Rong Wen, Sheng Bi

**Affiliations:** 1 Department of Ophthalmology, the First Affiliated Hospital of Harbin Medical University, Harbin, China; 2 Department of Neurology, the Fourth Affiliated Hospital of Harbin Medical University, Harbin, China; 3 Department of Neurology, the First Affiliated Hospital of Harbin Medical University, Harbin, China; 4 Perinatal Epidemiology, Sainte-Justine Hospital, University of Montreal, Montreal, Canada; 5 Central Laboratory, the First Affiliated Hospital of Harbin Medical University, Harbin, China; Beth Israel Deaconess Medical Center, Harvard Medical School, United States of America

## Abstract

Associations between interleukin 6 (IL-6) polymorphisms and Alzheimer’s disease (AD) remain controversial and ambiguous. The aim of this meta-analysis is to explore more precise estimations for the relationship between IL-6-174 G/C and -572 C/G polymorphisms and risk for AD. Electronic searches for all publications in databases PubMed and EMBASE were conducted on the associations between IL-6 polymorphisms and risk for AD until January 2012. Odds ratio (OR) and 95% confidence intervals (CIs) were calculated using fixed and random effects models. Twenty-seven studies were included with a total of 19,135 individuals, involving 6,632 AD patients and 12,503 controls. For IL-6-174 G/C polymorphism, the combined results showed significant differences in recessive model (CC vs. CG+GG: OR = 0.65, 95%CI = 0.52–0.82). As regards IL-6-572 C/G polymorphism, significant associations were shown in dominant model (CG+GG vs. CC: OR  = 0.73, 95% CI = 0.62–0.86) and in additive model (GG vs. CC, OR  = 0.66, 95% CI = 0.46–0.96). In conclusion, genotype CC of IL-6-174 G/C and genotype GG plus GC of IL-6-572 C/G could decrease the risk of AD.

## Introduction

As a major cause of cognitive decline in the elderly, Alzheimer’s disease (AD) currently affects 20–30 million individuals worldwide. It is estimated that the number of people likely to be affected will be triple over the next 50 years throughout the world. [1], [2] Although AD is a disease associated with several risk factors such as a series of epigenetic, genetic, endocrine, and external environmental factors, an increasing number of experimental evidences are suggesting a possible involvement of chronic inflammation in onset and progression of this disease. Interleukin-6 (IL-6) is a potent proinflammatory cytokine produced by diverse kinds of cells such as leukocytes, adipocytes, endothelial cells, fibroblasts, and myocytes. [3] It is associated with accumulation of acute phase proteins in neuritic plaques and amyloid precursor protein (APP) synthesis. [4] IL-6 gene, which maps to chromosome 7p21, has been postulated to be a good candidate genetic risk factor for AD. [5].

Two independent variants in the promoter region of IL-6 promoter (−174 G/C and −572 C/G) have been detected. Previous studies concerning association between IL-6-174 G/C polymorphism and risk of AD are limited and rather conflicting. The second IL-6 polymorphic exchange −572 C/G (which is identical to −634 C/G) is not commonly analyzed as −174 G/C. However, previous findings suggested that −572 C/G polymorphism might affect the transcription rate of the IL-6 gene, and furthermore influence plasma levels of acute proteins such as fibrinogen and C-reactive protein. [6], [7] Recently, five previous studies were also frequently performed on the effect of IL-6-572 C/G polymorphism on AD, but the results were conflicting.[8]–[12] Two previous meta-analyses [13], [14] regarding IL-6-174 G/C polymorphisms and AD have summarized the findings from certain studies, but a number of important studies were missed. In addition, there is no meta-analysis on −572 C/G polymorphisms. Given the importance of clarifying the potential role of IL-6 gene variants in AD and given the extensive and diverse body of evidence available, we conducted a comprehensive systematic and quantitative review of the evidence on the associations between −174 G/C and −572 C/G polymorphisms of the IL-6 gene and the risk of AD.

## Methods

### Literature Search

Two reviewers independently searched studies on the associations between IL-6 polymorphisms and AD. Published studies were identified through a computerized search of PubMed, MEDLINE, EMBASE, and the Cochrane Library in any language up to January 2012. The keywords were as follows: Alzheimer’s disease, interleukin and polymorphism or variant or genotype or SNP. The references of all identified publications were searched for additional studies, and the PubMed option “Related Articles” was also used to search for potentially relevant papers. We only included published articles written in English.

### Study Selection

Two reviewers independently identified potential relevant studies and evaluated each trial based on predetermined eligibility criteria. Studies were included if they met the following criteria: (1) the study reported original data from case-control studies; (2) the outcome had to be AD; (3) at least two comparison groups (AD patient group vs. control group) and the number of subjects possessing genotype in the AD and control groups were available; and (4) in the case of multiple publications from the same study group, the most complete and recent results were used.

### Data Extraction

After excluding the overlap studies and including the additional ones, this meta-analysis covered a total of 22 articles on IL-6-174 G/C polymorphism and 5 articles on IL-6-572 C/G polymorphism. Two authors extracted the data independently and in duplicate. The following data were extracted from the eligible studies: the first author’s last name, year of publication, country of origin, ethnicity, and numbers of genotyped cases and controls. Any disagreement was adjudicated with a third author.

### Statistical Analysis

The strength of the association between the IL-6 polymorphism and AD was measured by odds ratio (ORs) with 95% confidence intervals (CIs). We explored the allele comparison, as well as dominant model and recessive model. Heterogeneity among studies was examined with *I^2^* statistic interpreted as the proportion of total variation contributed by between-study variation. If there was a statistical difference in terms of heterogeneity (I^2^>50%, *P*<0.05), a random-effect model was selected to combine the data. Otherwise, a fixed-effect model was employed. The significance of the pooled OR was determined by the Z-test, and two-tailed *P*<0.05 was considered as statistically significant. Relative influence of each study on pooled estimates was assessed by omitting one study at a time for sensitivity analysis. Evidence of publication bias was determined by visual inspection of the funnel plot. All statistical analyses were performed with Revman 5.0 (Copenhagen: The Nordic Cochrane Centre, The Cochrane Collaboration, 2008).

## Results

### Eligible Studies

**Figure 1:**
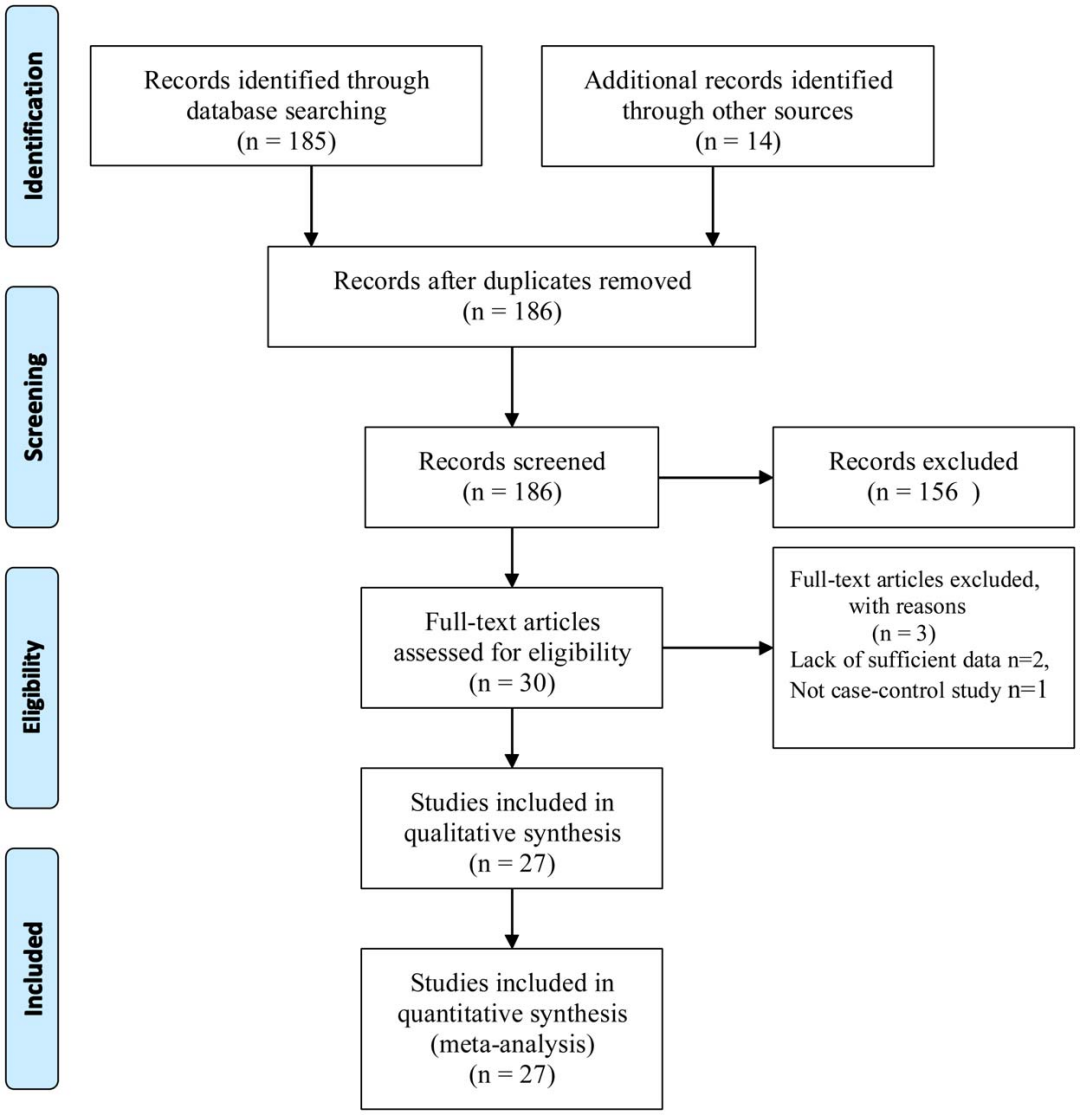
PRISMA Flow Diagram.

In this article, the associations of IL-6 polymorphisms with AD susceptibility were investigated using meta-analysis in a wide range of populations (**Figure 1**). Twenty-seven case-control studies (22 studies on IL-6-174 G/C polymorphism[15]–[36] and 5 studies on IL-6-572 C/G studies polymorphism[8]–[12]) met all of the inclusion criteria and were included in the review. The detailed characteristics of these studies are presented in **Table 1**. No evidence of publication biases were observed in Funnel plots (available upon request). Totally 4,280 AD patients and 8,788 controls for IL-6-174 G/C polymorphism and 2,352 AD patients and 3,715 controls for IL-6-572 C/G polymorphism were included in the analyses. Among included studies, five studies[12], [15]–[18] only reported GG and GC genotypes vs. CC genotype and one [19] only reported GC and CC genotypes vs. GG genotype information in their paper. Among the included studies, 17 studies were on IL-6-174 G/C allele contrast and additive model, 19 studies were on IL-6-174 G/C dominant genetic model and 20 studies were on IL-6-174 G/C recessive model. Though the number of studies on IL-6-572 C/G polymorphism was small, they were also included for a comprehensive evaluation in this study.

**Table 1:** Main characteristics of selected studies.

Author	Year	Country	Ethnicity	Cases	Controls
IL-6-174 G/C (rs1800795)					
Bagli et al.	2000	Germany	Caucasian	102	351
Bhojak et al.	2000	USA	Caucasian	464	337
Pola et al.	2002	Italy	Caucasian	124	134
Shibata et al.	2002	Japan	Asian	128	83
Faltraco et al.	2003	Japan	Caucasian	101	133
Licastro et al.	2003	Italy	Caucasian	332	393
Arosio et al.	2004	Italy	Caucasian	59	65
Capurso et al.	2004	Italy	Caucasian	168	220
Depboylu et al.	2004	Germany	Caucasian	113	108
Zhang et al.	2004	UK	Caucasian	356	434
Koivisto et al.	2005	Finland	Caucasian	65	542
Vural et al.	2009	Turkey	Caucasian	101	138
Capurso et al.	2010	Italy	Caucasian	149	298
Mansoori et al.	2010	India	Asian	74	113
Combarros et al.*	2005	Spain	Caucasian	234	197
Fontalba et al.*	2009	Spain	Caucasian	239	165
Infante et al.*	2004	Spain	Caucasian	232	201
Oijen et al.	2006	Netherlands	Caucasian	483	4069
Paradowski et al.	2008	Poland	Caucasian	51	36
Klimkowicz-Mrowiec et al.	2010	Poland	Caucasian	361	200
Papassotiropoulos et al. *	1999	UK	Caucasian	102	351
Mateo et al. #	2005	Spain	Caucasian	242	220
IL-6-572 C/G (rs1800796)					
Eriksson et al.	2011	USA	Caucasian	1255	2363
He et al.	2010	China	Asian	318	324
Nishimura et al.	2004	Japan	Asian	172	163
Wang et al.	2010	China	Asian	341	421
Chen et al.*	2012	Taiwan	Mix	266	444

* only reported GG and GC genotypes vs. CC genotype information.

# only reported GC and CC genotypes vs. GG genotype information.

### Quantitative Synthesis

#### Allele comparison

The association between the IL-6 polymorphisms and AD were showed in **Table 2** and **3**. For IL-6-174 G/C allelic contrast, the C allele was not associated with AD (C vs. G: OR  = 0.95, 95% CI = 0.83–1.10, *P* = 0.53). There was a significant difference between-study heterogeneity (I^2^ = 67%). No significant associations were also found for IL-6-572 C/G allele (G vs. C: OR  = 0.87, 95% CI = 0.69–1.10, *P* = 0.24). Heterogeneity was detected (I^2^ = 68%).

**Table 2:** Stratified analyses of the IL-6-174 G/C polymorphisms on AD risk.

Outcome or Subgroup	N	Cases/total	I^2^(%)	OR (95%CI)	P
C vs. G	17	6462/24902	67	0.95 (0.83, 1.10)	0.53
CC vs. GG	17	1678/6598	67	0.87 (0.65, 1.18)	0.38
CC vs. CG+GG	20	3997/13719	65	0.65 (0.52, 0.82)	**0.0003**
CC+GC vs. GG	19	3528/13267	90	1.01 (0.74, 1.38)	0.97

**Table 3:** Stratified analyses of the IL-6-572 C/G polymorphisms on AD risk.

Outcome or Subgroup	N	Cases/total	I^2^(%)	OR (95%CI)	P
G vs. C	4	4182/10720	68	0.87 (0.69, 1.10)	0.24
GG vs. CC	4	1673/4351	24	0.66 (0.46, 0.96)	**0.03**
CG+GG vs. CC	5	2352/5798	0	0.73 (0.62, 0.86)	**0.0002**
GG vs. CG+CC	4	2086/5357	60	0.82 (0.53, 1.25)	0.35

#### Genotype comparison

**Figure 2:**
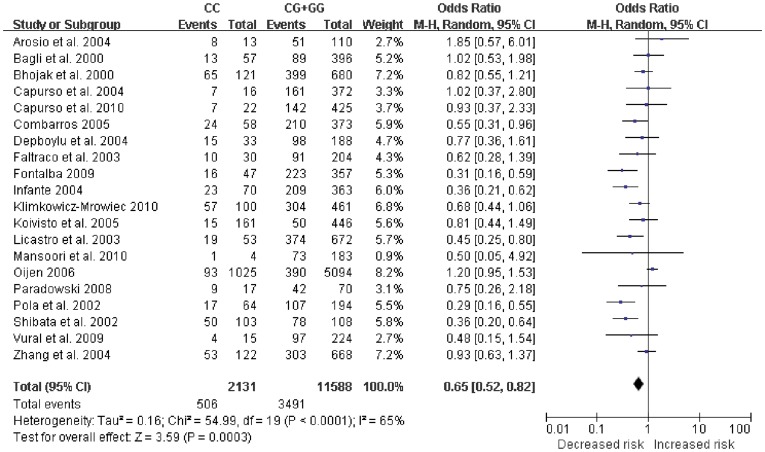
Forest plot of AD risk associated with IL-6-174 G/C polymorphism at recessive model (CC genotypes vs. CG+GG genotype). The squares and horizontal lines correspond to the study-specific OR and 95% CI. The area of the squares reflects the weight (inverse of the variance). The diamond represents the summary OR and 95% CI.

**Figure 3:**
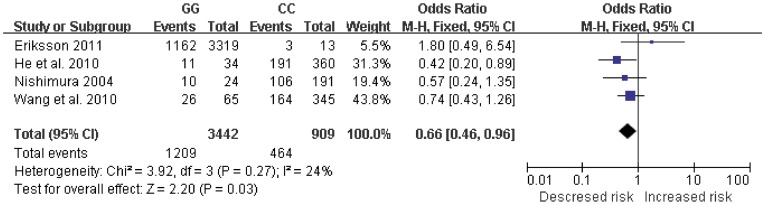
Forest plot of AD risk associated with IL-6-572 C/G polymorphism at additive model (GG genotype vs. CC genotype). The squares and horizontal lines correspond to the study-specific OR and 95% CI. The area of the squares reflects the weight (inverse of the variance). The diamond represents the summary OR and 95% CI.

**Figure 4:**
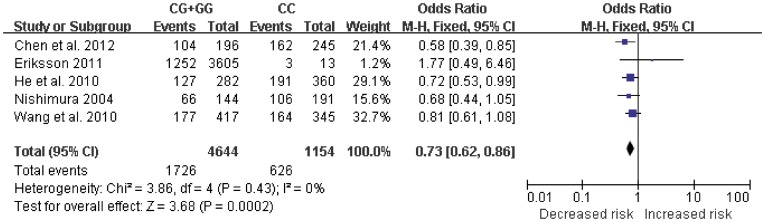
Forest plot of AD risk associated with IL-6-572 C/G polymorphism at dominant model (CG+GG genotype vs. CC genotype). The squares and horizontal lines correspond to the study-specific OR and 95% CI. The area of the squares reflects the weight (inverse of the variance). The diamond represents the summary OR and 95% CI.

The genotype frequency of the IL-6 polymorphisms between case and control groups were presented in **Table 2** **and** **3**. For IL-6-174 G/C polymorphism, the combined results based on all studies showed the evidence of an association between the decreased risk of AD and the variant genotypes in recessive model (CC vs. CG+GG: OR  = 0.65, 95% CI = 0.52–0.82, *P* = 0.0003, **Figure 2**). There was significant heterogeneity (I^2^ = 65%). No associations were found in dominant model or additive model. For IL-6-572 C/G polymorphism, significant decreased risk was found in additive model (GG vs. CC: OR  = 0.66, 95% CI = 0.46–0.96, **Figure 3**) and dominant model (CG+GG vs. CC: OR  = 0.73, 95% CI = 0.62–0.86, **Figure 4**). No heterogeneity was found. No associations were found in recessive model.

### Sensitivity Analysis

According to sensitivity analysis, we found that there was no substantial modification of our estimates after exclusion of individual studies (data not shown), indicating that the results of this meta-analysis were stable.

## Discussion

The main finding of this meta-analysis is that genotype CC of IL-6-174 G/C and genotype GG plus CG of IL-6-572 C/G are potential protective factors for developing and progressing AD.

The IL-6 gene polymorphisms are widely investigated in relation to the risk of AD. However, the results from these studies were ambiguous, for their small sample size and unified ethnicity. Although two meta-analysis studies have focused on −174 G/C polymorphism recently, none of them could give us results with all publications. [13], [14] Dai et al [13] performed a meta-analysis based on 18 studies on the relationship between IL-6-174 G/C and AD, and the combined results showed significant differences in recessive model (CC versus GC + GG: OR  = 0.70, 95%CI = 0.54–0.90). Han et al [14] showed that a borderline statistically significant association between the IL-6-174G/C polymorphism and AD risk in Caucasians (GG vs. CC: OR = 1.35, 95%CI = 1.06–1.72; GG/GC vs. CC: OR = 1.27, 95%CI, 1.05–1.53, respectively) on the basis of 14 studies on Caucasian. To the best of our knowledge, there is no meta-analysis to explore −572 C/G polymorphism in development and progression of AD. To achieve a more reliable and comprehensive conclusion on both variants, our comprehensive review covered 27 studies, including 22 on IL-6-174 G/C polymorphism[15]–[36] and 5 on −572 C/G studies polymorphism[8]–[12], versus 14 to 18 studies in previous reviews. With 6,632 AD patients and 12,503 controls for IL-6-174 and -572 polymorphisms investigations included, the results from our studies consent to previous meta-analyses and may confirm that the IL-6 −174G/C polymorphism may be a protective factor for the development of AD.

Because heterogeneity was found among the studies, we employed random-effect model. Then, a sensitivity analysis was performed by removing one study for each time and re-running the model to determine the effect on the overall estimate. The estimates changed quite little, strengthening the results from this meta-analysis. No publication bias was shown, also strengthening our results.

As inflammation is involved in the pathogenesis of AD, IL-6 has been implicated in both the development and progression through accumulation of acute phase proteins in plaques and elevation of amyloid precursor protein synthesis in experimental and human AD. [4] Since the −174 C allele and −572 G allele in the promoter region of IL-6 gene were reported to reduce IL-6 gene expression and IL-6 levels in the blood and brain from AD [8], [23], investigations on these and related variants have obtained a steady rise in AD studies during recent decades. Our findings showed that IL-6 polymorphism was associated with a decreased risk of AD, which may confirm the biologically plausible described above.

This meta-analysis has pooled all the available results from the case–control studies, which has significantly increased the statistical power. However, some limitations of this meta-analysis should be acknowledged. First, AD is a complex disease that results of combined effects of multifactor, including inherited and environmental factors. Some environmental factors may strongly influence the development of AD. Lacking of considering these factors may affect the significance for the independent role of IL-6 polymorphisms in AD development. Second, the network of cytokines is complex and variants of other pro-inflammatory cytokines may exert their complex and interacting functions with each other. No regard of these factors may cause serious confounding bias. Third, the number of current studies on IL-6-572 C/G is relative small. Thus, investigations involving more subjects of different races are needed to confirm the effect and then another meta-analysis should be necessary for a more reliable evaluation on their associations. Fourth, the heterogeneity observed in the relationship between −174 G/C polymorphism and AD, even when analyzed by ethnicity and a sensitivity analysis. Finally, we reviewed only published studies.

In conclusion, this meta-analysis detected significant associations between IL-6-174 G/C and −572 C/G polymorphisms and AD. Genotype CC of IL-6-174 G/C and genotype GG plus CG of −572 C/G could decrease the risk of AD. To better understand the potential mechanism for AD in humans, large well-designed cohort studies are needed to confirm these associations and further researches should be carried out to explore the effect of genetic networks, environmental factors, individual biological characteristics and their mutual interactions.
